# Evaluation of *ALK* Rearrangement in Chinese Non-Small Cell Lung Cancer Using FISH, Immunohistochemistry, and Real-Time Quantitative RT- PCR on Paraffin-Embedded Tissues

**DOI:** 10.1371/journal.pone.0064821

**Published:** 2013-05-31

**Authors:** Yun-Gang Zhang, Mu-Lan Jin, Li Li, Hong-Ying Zhao, Xuan Zeng, Lei Jiang, Ping Wei, Xiao-Li Diao, Xue Li, Qing Cao, Xin-Xia Tian

**Affiliations:** 1 Department of Pathology, Beijing Chao-Yang Hospital, Capital Medical University, Beijing, P. R. China; 2 Department of Pathology, Peking Union Medical College Hospital, Peking Union Medical College & Chinese Academy of Medical Sciences, Beijing, P. R. China; 3 Department of Pathology, Peking University Health Science Center, Beijing, P. R. China; National Taiwan University Hospital, Taiwan

## Abstract

Patients with ALK gene rearrangements often manifest dramatic responses to crizotinib, an ALK inhibitor. Accurate identification of patients with ALK-positive non-small cell lung cancer (NSCLC) is essential for the clinical application of ALK-targeted therapy. However, assessing *EML4-ALK* rearrangement in NSCLC remains challenging in routine pathology practice. The aim of this study was to compare the diagnostic accuracy of FISH, immunohistochemistry (IHC), and real-time quantitative RT-PCR (QPCR) methodologies for detection of *EML4-ALK* rearrangement in NSCLC and to appraise immunohistochemistry as a pre-screening tool. In this study, a total of 473 paraffin-embedded NSCLC samples from surgical resections and biopsies were analyzed by IHC with ALK antibody. *ALK* rearrangement was further confirmed by FISH and QPCR. ALK protein expression was detected in twenty patients (20/473, 4.2%). Of the 20 ALK-positive cases by IHC, 15 cases were further confirmed as *ALK* rearrangement by FISH, and 5 cases were not interpretable. Also, we evaluated 13 out of the 20 IHC-positive tissues by QPCR in additional to FISH, and found that 9 cases were positive and 2 cases were equivocal, whereas 2 cases were negative although they were positive by both IHC and FISH. The ALK status was concordant in 5 out of 8 cases that were interpretable by three methods. Additionally, none of the 110 IHC-negative cases with adenocarcinoma histology showed *ALK* rearrangements by FISH. Histologically, almost all the *ALK*-rearranged cases were adenocarcinoma, except that one case was sarcomatoid carcinoma. A solid signet-ring cell pattern or mucinous cribriform pattern was presented at least focally in all ALK-positive tumors. In conclusion, our findings suggested that *ALK* rearrangement was associated with ALK protein expression. The conventional IHC assay is a valuable tool for the pre-screening of patients with *ALK* rearrangement in clinical practice and a combination of FISH and QPCR is required for further confirmation.

## Introduction

Lung cancer remains the leading cause of cancer-related death worldwide [1], [2], despite of improvements in detection methods and treatments. Among non-small cell lung cancer (NSCLC), accounting for approximately 85% of all lung cancers, adenocarcinoma is the most common type of lung cancer in both men and women [2]. Currently, efforts are devoted to development of molecules against specific targets for specific tumor types. With continued improvement in our understanding of the molecular basis of lung cancer, a number of targeted therapies for NSCLC are being evaluated or developed. These therapies include angiogenesis inhibitors and signaling transduction inhibitors such as the epithelial growth factor receptor (EGFR)-targeted therapies [3]. Therefore, the identification of the key oncogenes for lung cancer is a very important step toward the development of novel molecular-targeting agents.

Recently, activation of the anaplastic lymphoma kinase (ALK) gene in lung cancer by fusion to echinoderm microtubule-associated protein-like4 (EML4) or other gene partners (such as *TFG* and *KIF5B*) has been reported [4], [5], [6]. The ALK gene was initially characterized as a fusion partner of the NPM-ALK oncogene in anaplastic large cell lymphoma [7], and is now recognized as the active component in multiple fusion proteins in a variety of cancers [8]. ALK is a transmembrane receptor with tyrosine kinase activities belonging to the insulin growth factor receptor superfamily, which is encoded on chromosome 2 (2p23). The various fusion partners of ALK mediate ligand-independent dimerization of ALK, resulting in constitutive kinase activity, and thus transmits anti-apoptotic and cell proliferation signals via KRAS and PI3K pathways [8]. In NSCLC, aberrant activation of ALK contributes to lung carcinogenesis after being fused with a number of other gene partners, most frequently echinoderm microtubule-associated protein 4 (*EML4*). EML4 gene is located on chromosome 2 (2p21) and is reversely oriented with *ALK*. *EML4-ALK* fusion might occur after a cleavage of the chromosome at a variable site and chromosome inversion, giving rise to different fusion isoforms [9]. *EML4-ALK* fusion occurs in a mutually exclusive fashion with *EGFR* or *KRAS* mutations and almost exclusively in adenocarcinoma. The presence of *EML4-ALK* is more likely in patients with certain demographic characteristics, such as never smoking status or younger age [10]. Most studies have discovered this genetic abnormality at a rate of 2–5% in the general population of patients with NSCLC [11], [12], [13], [14].

In previous clinical trials, Crizotinib, a dual ALK/MET kinase inhibitor, was shown to be dramatically effective in patients with NSCLC harboring ALK gene rearrangements [15]. Crizotinib was recently approved by the US FDA for the treatment of advanced *ALK*-positive NSCLC identified by fluorescence in situ hybridization (FISH) [16]. To ensure identification of patients most likely to benefit from Crizotinib, it is vital to develop robust and easy diagnostic methods to detect ALK gene rearrangement when screening patients for treatment with crizotinib. Although ALK FISH has become the gold standard for detecting *ALK* rearrangement in NSCLC, it can be technically challenging and costly. Therefore, other diagnostic modalities remain to be explored, including immunohistochemistry (IHC) and reverse transcriptase-polymerase chain reaction (RT-PCR). RT-PCR is a highly sensitive technique for detection and quantification of RNA for *EML4-ALK*. It is also capable of typing the *EML4-ALK* variant by sequencing of the PCR product. However, because this methodology may not identify novel rearrangements involving previously uncharacterized *EML4-ALK* variants or unknown fusion partners and its process may be readily contaminated, its sensitivity and specificity remain to be validated. IHC has the advantage of being widely available, relatively easy to perform and retains morphological information, which allows confident assessment of aberrant genes in tumor cells. For this reason, ALK IHC seems suitable for a large-scale screening of patients with *ALK*-positive NSCLC.

The major challenge to using ALK IHC is the often low-level expression of ALK fusion proteins in *ALK*-rearranged NSCLC [16], which makes it necessary to develop more sensitive IHC-based methods. Several ALK antibodies have been reported in recent studies showing that IHC has high concordance with ALK FISH, including ALK1 monoclonal antibody from DAKO [17], 5A4 monoclonal antibody from Novocastra [18], and D5F3 monoclonal antibody developed by Cell Signaling Technology [19]. Nevertheless, large-scale studies are required to further validate concordance between IHC and FISH, and the development and evaluation of new antibodies with high specificity and sensitivity for ALK fusion proteins would be very welcome.

In this study, we examined ALK gene rearrangement using immunohistochemistry in NSCLC clinical samples, and correlated the results with those obtained by FISH and QPCR. In addition, we investigated clinicopathologic characteristics of NSCLC with ALK gene rearrangements. The present study was aimed at identifying useful information predicting ALK gene rearrangement and determining a diagnostic procedure that could be performed in routine practice.

## Materials and Methods

### Patients and Samples

This study was performed with the approval of the Clinical Ethics Committee of Beijing Chao-Yang Hospital, Capital Medical University. All patients provided written informed consent. Archival formalin-fixed paraffin-embedded tissues were obtained from 473 patients who had undergone treatment of primary lung cancer between 2006 and 2011 at the Beijing Chao-Yang Hospital of Capital Medical University in China. Cases were randomly selected among patients diagnosed as NSCLC by histologic examination of specimens obtained by surgical resection and biopsy. No patients had received chemotherapy before surgery and biopsy. For all cases, we reviewed age at diagnosis, gender, smoking history, and location of primary tumor, which were obtained from medical records. According to the7th edition TNM staging manual for lung cancer published by AJCC in 2010, all cases were reviewed and staged. All available histology slides from each case were reassessed, and the histological type and grade of differentiation were confirmed according to the criteria of the World Health Organization Classification of lung Tumors and the ERS/ATS/IASLC multidisciplinary classification of lung adenocarcinoma [20]. For each case, the representative tissue block containing most viable tumor cells was selected by two pathologists. The stage of the disease was postoperative pathologic stage. The stage IV diseases included cases who received biopsies by open lung or video-assisted thoracoscopic surgery for diagnosis. The clinicopathological data are summarized in Table 1.

**Table 1:** Clinicopathological characteristics of patients with Non–Small-Cell Lung Cancer in this study.

Variable	Group	No. (%)
**Age**	Median	59
	Range	21–84
**Gender**	Male	314(66.4)
	Female	159(33.6)
**Smoking history** ^*^	Never or light smoker	180(38.2)
	Smoker	293(61.8)
**Histological type**	Adenocarcinoma	341(72.1)
	Squamous cell carcinoma	112(23.7)
	Adenosquamous carcinoma	4(0.8)
	sarcomoid carcinoma	9(1.9)
	large cell carcinoma	3(0.7)
	salary type	4(0.8)
**Stage** ^#^	IA	80(16.9)
	IB	86(18.2)
	IIA	76(16.1)
	IIB	28(5.9)
	IIIA	89(18.8)
	IIIB	16(3.4)
	IV	98(20.7)
Total		473(100)

Note: * Never smokers have smoked <100 cigarettes in their lifetime; light smokers have smoked <10 pack years; and smokers have smoked >10 pack years. ^#^ Pathological stage represents stage at the time of resection or biopsy. Stage was determined according to the 7th Edition of the AJCC/UICC TNM Cancer Staging Manual; however, patients with malignant pleural effusions were classified as stage IV.

### ALK Detection with Immunohistochemistry

For each case, a representative formalin-fixed paraffin-embedded (FFPE) tissue block was chosen and sectioned at 4-µm thickness for immunostaining. Briefly, after deparaffinization and rehydration, the slides were heated for antigen retrieval in pressure cooker for 3 minutes at 100°C in 0.01mol/L Tris-EDTA buffer (pH 9.0). Endogenous peroxidase activity was blocked by incubating the slides in 3% hydrogen peroxide in absolute methanol for 15minutes, and nonspecific binding was blocked with 10% goat serum for 15 minutes. A rabbit monoclonal antibody to ALK (Clone SP8; Thermo Fisher Scientific, Fremont CA, USA) was used as the primary antibody at a dilution of 1∶100. After overnight incubation with the ALK antibody at 4°C, the slides were incubated with an anti-rabbit, horseradish peroxidase-labeled polymer secondary antibody from the DAKO Envision +™ System (Dako Corporation). Immunoreactivity was visualized with 3,3′-diaminobenzidine (Dako Corporation, Carpinteria, CA). Finally, the sections were counterstained with hematoxylin and mounted. Positive control sections from known ALK-positive ALCL cases were included in each staining batch. Normal lymph node tissue was used as a negative control. For blank control, all incubation steps were identical except that rabbit nonimmune IgG serum was used rather than the primary antibody (ALK).

We determined the scoring criteria during a preliminary evaluation using a multi-headed microscope in order to reach a consensus. Staining results were then interpreted by two of the authors independently, without prior knowledge of the clinicopathological parameters. Discordant cases were reviewed and agreed upon before data were statistically analyzed. For each sample, at least five fields (×400) and more than 500 cells were analyzed. For the ALK stains, only unequivocal cytoplasmic staining was considered as a positive reaction. The number of immunopositive cells was semiquantitatively estimated: no positive cells (−); <50% of the tumor cells staining positive (+); 50%–75% of tumor cells staining positive (++); >75% of tumor cells staining positive (+++).The staining intensity was graded on a scale from 0 to 3+ (0, negative;1+, weak; 2+, moderate; 3+, intense), similarly to the previously described protocols [17], [18].

### Fluorescence In Situ Hybridization (FISH)

FISH was performed on formalin-fixed, paraffin-embedded (FFPE ) tumor tissues using the Vysis LSI ALK Dual Color, Break Apart Rearrangement Probe (Abbott Molecular, Abbott Park, Illinois, USA). The ALK break-apart probe set includes two DNA probes labeled with Spectrum Orange and Spectrum Green that respectively hybridize to the band 2p23 on opposite sides flanking the breakpoint of the ALK gene. Assay was performed according to the manufacturer’s instructions. Briefly, 4-µm-thick sections from FFPE tissue blocks were deparaffinized and dehydrated, then the sections were treated with Vysis pretreatment reagent (Abbott Molecular) and reacted with protease solution (Abbott Molecular). The ALK break-apart probe mixture was applied to the entire tissue, and they were co-denatured at 73°C for 3 minutes. The slides were then hybridized overnight in a humidified chamber at 37°C. After washing, nuclei were counterstained with 4′,6-diamidino-2-phenylindole (DAPI). Sections were analyzed under a fluorescence microscope equipped with a triple-pass filter (DAPI/Green/Orange). A repeated analysis of some samples was carried out due to poor hybridization signals and/or poor tissue morphology. Positive and negative control slides were included in each run of FISH test.

### FISH Interpretation

According to the manufacturer′s specifications, FISH slides were evaluated without knowledge of the IHC results for ALK. The two technicians analyzed each FISH slide, scanning the entire tissue section and scoring 50 representative nuclei, which were clearly identified and contained unequivocal signals. Nuclei containing signals of only one color should not be enumerated. The results of each technician were not combined until study completion to minimize any bias in scoring. The cell was regarded as positive, when a nucleus had at least one set of broken apart signals, or had a single red signal (deleted green signal) in addition to fused and/or broken apart signals. The distance between two separated red and green signals was estimated using the two times of biggest signal size. The samples were considered positive if more than 25 out of 50 tumor cells were positive, and negative if less than 5 tumor cells were positive. The sample with 5–25 positive tumor cells was considered equivocal, and then was evaluated by a second technician. The first and second cell count readings were added together and a percent was calculated out of 100 cells. If the average percent of the positive cells was 15% or more, the sample was considered positive. Otherwise, it was considered negative for *ALK* rearrangement.

### Real-time Quantitative RT-PCR(QPCR)

Total RNA was extracted from freshly cut FFPE tissue sections using the RNeasy kit (Qiagen). Briefly, tumor area was identified through hematoxylin-eosin staining and tissue from this area on unstained sections was scraped for RNA extraction. After deparaffinization and lysis steps, the total RNA was purified with an RNeasy MinElute spin column. Genomic DNA was removed with RNase-Free DNase I (Qiagen). Before RNA amplification, the integrity and purity of RNA was estimated by denaturing agarose gel electrophoresis and A260/A280 measurement.

Real-time PCR amplification was performed using the AmoyDx™ EML4-ALK Fusion Gene Detection Kit (Amoy Diagnostics, Xiamen, China) according to the manufacturer’s recommendations. Briefly, 0.1–5 µg of total RNA was reverse transcribed to cDNA using M-MLV Reverse Transcriptase(invitrogen). Six microliters of cDNA were then used as template for a 25-µl reaction for the real-time PCR detection of the EML4-ALK fusion transcripts using the EML4-ALK Reaction Master Mixes and an ABI 7500 cycler (Applied Biosystems). The EML4-ALK Fusion Gene Detection Kit employs novel, proprietary primers to specifically amplify the target gene sequence involving nine known EML4-ALK fusion transcript variants, including E13;A20, E6a/b;A20, E20;A20, E15;A20, E14;A20, E18;A20, E2;A20, E17;A20. The amount of target cDNA was measured after each cycle in the data capture phase using a novel fluorescent probe. The quality of the synthesized cDNA was verified during the same run by amplification of the reference gene (β-actin, *ACTB*). The EML4-ALK fusion gene and *ACTB* assays were labeled with FAM. A positive and negative control was included in each real-time PCR run. As defined by the manufacturer’s instructions, QPCR assay for EML4-ALK fusion gene was considered positive, if the sample Ct value <30. For the negative samples, real-time RT-PCR assay were repeated twice.

### Statistical Analyses

The statistical analyses were carried out by using the SPSS for Windows 13.0 program (SPSS Inc., Chicago, IL). To analyze correlations between *ALK* status and clinical-pathologic variables, we used the χ2 test or Fisher’s exact test, where applicable. Probability values of <0.05 were considered significant in the analyses.

## Results

### Clinicopathogical Features of Tumors

In the present study, we analyzed a panel of 473 NSCLC samples, comprising 375 cases from surgically resected tumors, and 98 cases from biopsies. No patients received chemotherapy at the time of diagnosis. As are summarized in Table 1, the patients composed of 314 (66.4%) men and 159 (33.6%) women with a median age 59 years (from 21 to 84 years). Histologically, 341 (72.1%) was adenocarcinoma, 112 (23.7%) squamous cell carcinoma, and 20 (4.2%) other types. The pathologic stage was I in 166 (35.1%), II in 104 (22%), III in 105 (22.2%), and IV in 98 (20.7%) cases.

### ALK Protein Expression by IHC

**Figure 1:**
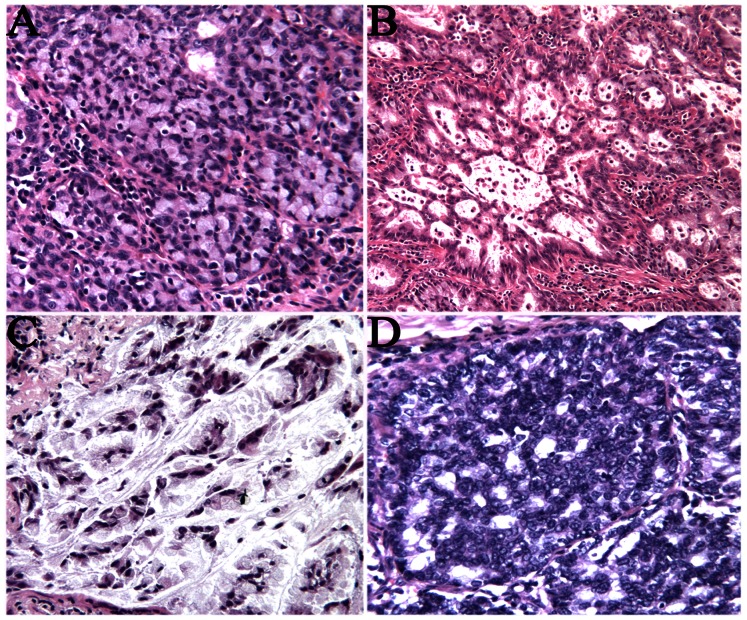
Comparison between ALK immunohistochemical staining and fluorescence in situ hybridization signals. **A, C, E, G:** ALK immunostainings (original magnification ×200). **A** and **C**: intense and moderate cytoplasmic stainings (score = 3 and 2 respectively), **E**: weak cytoplasmic stainings (score = 1 ) and **G**: no staining (score 0). **B**, **D**, **F** and **H**. FISH analysis for *ALK* using a dual-color ALK break-apart probe. **B**, **D** and **F** show FISH sigals in the ALK-rearranged tumors, and the proportion of cells with ALK positive signals was 49%(49/100), 38%(38/100), 56%(28/50), respectively. Rearranged *ALK* (**B**, **D**,and **F**) is indicated by splitting of red and green signals or single red signals.**H** shows FISH sigals in a non-rearranged tumor**.** the proportion of cells with ALK positive signals was 3%(3/100). Non-rearranged *ALK* (H) shows fusion or adjacent red and green signals.

All the ALK-positive cases exhibited a diffuse and cytoplasmic staining pattern in tumor cells, and no immunostaining was observed in normal lung bronchial epithelium, alveolar pneumocytes, alveolar macrophages, mesenchymal tissue, and inflammatory cells in the adjacent lung tissues (Figure 1). Twenty cases out of 473 were ALK-positive, representing 4.2% of all cases studied by IHC, which included score of 3 seen in 14 cases with a granular intense cytoplasmic staining (Figure 1-A), score of 2 in 5 cases with a moderate cytoplasmic staining (Figure 1-C), score of 1 in 1 case with a faint cytoplasmic staining (Figure 1-E) and score of 0 in 453 cases (Figure 1-G). No significant intratumoral staining heterogeneity was observed, although signet-ring cells tended to exhibit weaker staining than the other cells, perhaps because cytoplasmic ALK protein was diminished by a large volume of mucus material. There was no apparent correlation between ALK gene rearrangement by FISH and the intensity of immunostaining.

### ALK Rearrangement Assessed by FISH

ALK rearrangement was assessed using FISH in 110 IHC-negative cases with adenocacinoma histology and 20 IHC-positive cases. Among the 20 IHC-positive cases, 15 cases were confirmed as *ALK* rearrangement by ALK FISH, 5 cases were not interpretable, due to the technical artifacts such as signal loss that possibly resulted from inappropriate fixation or loss of tumor tissue. None of the 110 IHC-negative cases showed *ALK* rearrangement by FISH. Representative images of ALK FISH are shown in Figure 1-B, D, F, H. There were two positive *ALK* rearrangement patterns. One was the break-apart (BA) pattern with one or two separated red and green signals, and another was isolated red signal pattern without corresponding green signal. The ALK FISH-negative cases showed two fusion signals or close proximity of the red and green signals.

### Correlation between ALK IHC and FISH

In the present study, 130 cases were analyzed by both IHC and FISH. Concordance between IHC and FISH is shown in Table 2. Without considering 5 cases by FISH were not interpretable, the sensitivity and specificity of IHC in the 125 cases, in comparision with FISH, were 100% and 100%, respectively. Thus, this study showed that there was high concordance in the assessment of *ALK* rearrangement between IHC and FISH.

**Table 2:** Concordance between IHC and FISH assays from 130 cases detected by both two methods.

	FISH(+)	FISH(−)	FISH uninterpretable
IHC+ (N = 20)	15	0	5
IHC- (N = 110)	0	110	0

### EML4-ALK Fusion Gene by QPCR

We then tested 13 IHC-positive cases by QPCR. The EML4-ALK fusion genes were detected in 9 out of 13 cases. They included *EML4-ALK*variant 1, 2, 3 with exon 13 of *EML4*, exon 20 of *EML4*, and exon 6a/b of *EML4* fused to exon 20 of *ALK*, respectively. There were 2 cases in which EML4-ALK fusion genes were not detected by QPCR, but *ALK* rearrangements were detected by FISH. In addition, the positive result for *EML4-ALK* in 2 cases could not recur in the repeated analysis. Of note, in one case, ALK FISH was uninformative for *ALK* rearrangement, but the case was reported positive by QPCR. This could probably be due to the paucity of the tumor cells. In this sample, a total of 30 representative tumor cell nuclei were counted, and only two tumor cells were positive.

### Clinicopathologic Characteristics of ALK-positive Patients

**Figure 2:**
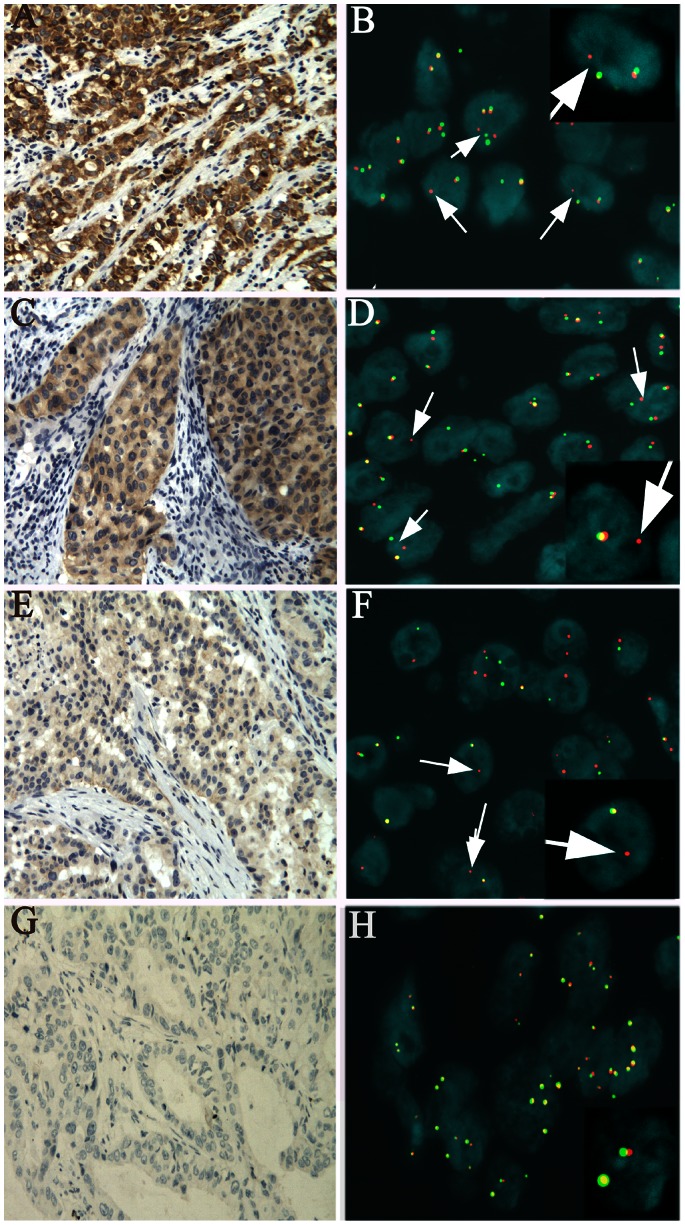
Histological features of ALK-rearranged lung adenocarcinomas. They showed solid signet-ring cell pattern (A), a cribriform structure (B.D), and abundant extracellular mucus (C).

A total of 473 resected and biopsied NSCLC samples were examined. ALK protein expression was evaluated in all cases by IHC. *EML4-ALK* translocations and/or *ALK* rearrangements were further confirmed in all ALK-immunopositive cases by FISH and/or QPCR. The relation between *ALK* rearrangement and clinicopathologic features is summarized in Table 3. *ALK* rearrangement was detected in 20 of 473 NSCLC cases. Of the 20 ALK-positive cases, 19 (95%) were adenocarcinomas, and 1 was sarcomatoid carcinoma which had a significant spindle cell and giant cell component. Histomorphologically, as shown in Table 4, the *ALK*-rearranged lung adenocarcinomas frequently showed solid signet ring cell pattern. A solid signet-ring cell pattern or mucinous cribriform pattern was presented at least focally in all ALK-positive tumors (Figure 2). As shown in Table 3, ALK-positive patients showed statistical difference in age (p = 0.003) and pathological stage (p = 0.024), compared with ALK -negative patients. Patients with ALK-positive lung adenocarcinoma were younger at diagnosis and had a higher stage, most commonly at stage IV. No appreciable relationship was demonstrated between ALK rearrangement and either patient gender or smoking history.

**Table 3:** Comparison of clinicopathologic parameters between ALK-positive and ALK-negative lung adenoarcinomas.

Variable	Group	ALK-positive	ALK-negative	P value
**Age**(years)	≤60	16	208	**0.003**
	>60	4	245	
**Gender**	Male	14	300	0.727
	Female	6	153	
**Smoking history**	Never or light smoker	7	173	0.774
	Smoker	13	280	
**Histological type**	Adenocarcinoma	19	322	**0.02**
	Others	1	131	
**Stage**	I	5	161	**0.024**
	II–III	6	203	
	IV	9	89	
**Total**		20	453	

**Table 4:** Characteristics of patients with ALK aberrations.

Case No.	sex	age	somoking	stage	Histomorplogy	ALK IHC	ALK FISH	QPCR
1	M	52	yes	IIA	sarcomoid carcinoma with cribriform pattern	2	NS	**+**
2	F	34	no	IB	solid pattern with mucin production and acinar pattern	3	+	−/+
3	M	21	yes	IV	solid signet-ring cell pattern	3	WS	−/+
4	M	24	yes	IV	solid signet-ring cell pattern and acinar pattern	3	?*	+
5	F	77	no	IA	acinar pattern	3	WS	+
6	F	38	no	IV	acinar predominant,with papillary and solid pattern	2	+	+
7	M	62	yes	IIIB	acinar and cribriform pattern	3	+	-
8	M	51	yes	IIIB	acinar and cribriform pattern with extracellular mucus	3	+	+
9	F	23	no	IV	acinar and cribriform pattern	3	+	ND
10	M	61	no	IA	acinar and cribriform predominant with solid signet-ring cell pattern	3	NS	+
11	F	30	no	IV	solid signet-ring cell pattern	1	+	+
12	M	37	yes	IV	solid pattern with mucin production	3	+	ND
13	F	68	no	IIIA	acinar and papillary predominant pattern, with solid signet-ring cell pattern	3	+	-
14	M	55	yes	IV	solid signet-ring cell pattern	3	+	+
15	M	53	yes	IIIA	papillary predominant with micropapillary and solid pattern	2	+	+
16	M	51	yes	IIIB	solid signet-ring cell pattern	3	+	ND
17	M	53	yes	IV	solid signet-ring cell pattern	2	+	ND
18	M	48	yes	IA	acinar predominant with papillary and solid pattern	3	+	ND
19	M	33	yes	IA	acinar predominant with papillary, cribriform and solid signet-ring cell pattern	3	+	ND
20	M	25	yes	IV	solid pattern with mucin production	2	+	ND

Abbreviations: NS: No FISH singals;WS:Weak FISH signals;ND:Not done.

* : Due to the paucity of tumor cells in this sample, the result by FISH was interpreted as uninformative and categorized as uninterpretable in statistic analyses.

## Discussion

Although the EML4-ALK fusion gene is a minor genetic abnormality in NSCLC [21], [22], the incidence of lung cancer is increasing in many countries, the absolute number of lung cancer patients harboring the EML4-ALK fusion gene is not trivial. Crizotinib, a dual MET/ALK tyrosine kinase inhibitor has been shown to be effective for the treatment of patients with ALK-positive NSCLC. Based on its efficacy and safety, crizotinib was recently approved by the US FDA for the treatment of patients with advanced ALK-positive NSCLC. However, the incidence of *ALK* rearrangement is relatively low, ranging from 2 to 5%. Even when an enriched population of NSCLC patients are selected on the basis of their predictive clinical characteristics, such as younger age, non-smoking status, and adenocarcinoma histology, it is difficult to identify the subsets of ALK-positive tumors. Therefore, efficient screening for patients with *ALK* –rearranged NSCLC is a crucial issue in clinical practice.

The ALK break-apart FISH assay has served as the companion dignostic test for establishing ALK positivity in clinical trials of crizotinib [15]. In theory, ALK FISH is capable of detecting any rearrangement involving *ALK*, regardless of the fusion partners or the *EML4-ALK* variants, on FFPE tissue which represents the most common method for processing and storing tumor specimens. However, from the technological and cost perspective, FISH for the routine large-scale detection of *ALK* rearrangement in NSCLC remains to be challenging. It is urgent to develop and validate more cost-effective methods for detecting this genetic aberration. Current diagnostic approaches to detect *ALK* rearrangement include immunohistochemistry (IHC), FISH and polymerase chain reaction (PCR)-based methods. However, sufficient details for an adequate comparison with FISH are currently lacking.

IHC is a rapid and affordable method preferred by pathologists for routine diagnosis. It can be performed successfully on a variety of histology and cytology specimens. Although the immunohistochemical technique does not directly detect the ALK fusion gene itself, based on the observation that ALK is not detectable in any normal tissues other than the brain [23], ALK-immunoreactivity is associated with upregulated expression of the gene, due to the altered promoter activity that is highly characteristic of an *ALK* inversion. Previous studies have shown that some commercially available ALK antibodies have relatively lower sensitivity in detecting ALK by IHC, possibly due to a weak transcriptional activity of the promoter-enhancer region of *EML4* that drives the expression of EML4–ALK. The D5F3 antibody (Cell Signalling Technology) is one of the most promising antibodies for the detection of *ALK* rearrangement in NSCLC [19]. However, further studies remain required to validate concordance between IHC and FISH in larger series of samples.

In the present study, we performed immunohistochemistry using monoclonal antibody (Clone SP8; Thermo Fisher Scientific, Fremont CA, USA), one commonly used, well-tested ALK antibody at the same dilution as used in ALCL, making its use more practical from a diagnostic laboratory perspective. This antibody recognizes a human p80 protein, identified as a hybrid of ALK gene and the nucleophosmin (NPM) gene resulting from the t(2;5)(p23;q35) translocation found in 30–50% of CD30+ large cell lymphomas. It also recognizes the full-length ALK protein. Our IHC method is based on a standard IHC method for routine pathology practice. We prolonged antigen retrieval time and incubated antibody overnight in 4°C to enhance sensitivity and specificity. The ALK-positive rate in our NSCLC cases (20/473, 4.2%) was comparable to the rate of EML4-ALK fusion transcript reported in the previous studies (2–5%). All the ALK-positive NSCLC cases by IHC showed ALK gene rearrangement by FISH and/or QPCR. On the other hand, among 473 NSCLC cases, we randomly selected 110 IHC-neagative cases with adenocarcinoma histology to evaluate *ALK* rearrangement by FISH, and found that there were no cases in which *ALK* gene rearrangement was detected. In contrast to a previous study [6], the IHC test with SP8 antibody showed higher specificity and sensitivity in our study. The ALK staining provided a very clean, easily interpretable immunoreactivity without disturbing background staining in the positive cases. This discrepancy presumably resulted from the different IHC procedure such as antigen retrieval and antibody incubation, or resulted from the variations in tissue processing in different laboratories. Our results remained to be confirmed in lager cohort studies.

RT-PCR of cDNA has been a commonly applied screening strategy for ALK gene rearrangements. By sequencing of the PCR product, the specific EML4-ALK variants expressed can be identified. However, novel ALK fusion partners will not be detected with such techniques. In recent studies, RT-PCR based detection of *ALK* rearrangement has been largely constrained to fresh or frozen tissue. Compared to multiplex RT-PCR, QPCR appears a perfectly suited method for the direct identification of fusion variant and detection of its expression level, because of its low cost and fast turnaround time. RT-PCR based detection of *ALK* rearrangement on FFPE tissues requires optimization of assay design and remains to be assessed in large cohort studies. In our study, we performed QPCR on FFPE tissues from 13 ALK-positive cases by IHC. 2 cases were negative for *EML4-ALK* by QPCR, but positive for *ALK* arrangement by FISH. Presumably, there were novel ALK fusion partners or novel *EML4-ALK* variant, while our assay design involved only nine known *EML4-ALK* variants. This remained to be validated by gene sequencing. In additional, degradation of RNA in FFPE tissue blocks may also lead to false-negative results. In our study, all the tested FFPE tissue blocks were 7–45 months-old when our detection work was performed. RNA quality and the absence of contamination with genomic DNA were verified by formaldehyde-agarose gel electrophoresis. We also quantified the level of β-actin as the endogenous RNA quality control. The required quality control including a no-template control, known ALK-positive and negative control, was performed throughout the entire procedure. Our results demonstrated QPCR on FFPE tissues was not efficient enough as a sole detection method, but it remained useful to identify the involved fusion variant when frozen material is not available.

We performed FISH, which is considered as the current gold standard for detecting *ALK* rearrangement on FFPE tissue blocks. In 5 out of 20 of ALK-positive cases by IHC, the ALK FISH results were not interpretable because of no or weak FISH signal and loss of tumor tissue. Presumably, higher interpretability should be obtained if using more recent samples. In one case positive for ALK by IHC and QPCR on FFPE tissue obtained by biopsy, ALK FISH was uninformative. This may be due to the paucity of tumor cells.

Our study was performed on FFPE tissues from surgically resected and biopsied samples. ALK-positive cases tended to have an advanced stage and their samples were obtained from biopsies. In daily clinical practice, we often obtain biopsy samples. It may be difficult to perform QPCR on FFPE tissues, because the amount of RNA extracted from such samples would probably not be sufficient enough for assay. Because each technique is associated with specific strengths and weakness for detecting *ALK* rearrangement, a combination of different detection methods may enhance chances to identify this genetic aberration. We propose that in routine practice, immunohistochemistry can be a pre-screening method, and FISH assay can be performed as a first confirmation method. Subsequently, QPCR can be immplemented in order to identify the specific *ALK* variant and detect its expression level. Such combination would be probably required to further investigate whether the *EML4-ALK* variant type or its expression level are correlated with the response of tumors to crizotinib in the future.

Thus, these results suggested that *ALK* rearrangement was associated with ALK immunoreactivity, and that our immunohistochemistry had high specificity as well as high sensitivity. The conventional IHC assay is a valuable tool for the prescreening of patients with *ALK* rearrangements in clinical practice and a combination of FISH and QPCR is required for further confirmation.
